# Ascending Aortic Thrombus With Peripheral Embolization

**DOI:** 10.7759/cureus.28766

**Published:** 2022-09-04

**Authors:** Nuno Maia Neves, Susana Carvalho Coelho, Natália Freitas Marto, Alexandra Bayão Horta

**Affiliations:** 1 Internal Medicine Department, Hospital da Luz Lisboa, Lisbon, PRT

**Keywords:** anticoagulation, mobile thrombus, thoracic aorta, ischemia, embolization, ascending aorta thrombus

## Abstract

Thoracic aortic mural thrombi are rare in clinical practice, especially in non-aneurysmatic or non-atherosclerotic vessels. They are typically located in the descending aorta, and less frequently in the aortic arch, abdominal aorta, and ascending aorta. Although they are a rare cause of arterial embolization, this is their main manifestation. We present the case of a 48-year-old man, with no cardiovascular risk factors or history of trauma, who presented with acute arterial ischemia of the right upper limb. From the initial investigation, we highlight the presence of a pedunculated mass in the distal portion of the ascending aorta with signs of instability. Due to the risk of additional embolization, the patient was submitted to urgent surgery, with excision of the aortic defect, implantation of a tubular prosthesis as well as thrombo-embolectomy of the right brachial artery. The etiological evaluation of mural aortic thrombi is challenging and implies the exclusion of some prothrombotic conditions known to predispose to arterial thrombosis. This is a rare case that emphasizes the importance of considering the aorta as a possible source of peripheral embolization, even when there is no significant atherosclerotic or aneurysmatic disease.

## Introduction

In the absence of significant atherosclerotic or aneurysmatic disease, mural aortic thrombi are uncommon. In many patients they are idiopathic; however, some prothrombotic conditions have been described as possible causes (malignancy, polycythemia, thrombocytosis, congenital or acquired hypercoagulable states, and primary tumors of aorta) [1-3], as well as genetic alterations of the aortic wall [4,5]. Of all segments of the aorta artery, its location in the ascending portion is the rarest [1]. Given the low frequency in quotidian clinical practice, the best therapeutic approach is controversial, depending on several factors [1,5,6]. However, pedunculated or large mural thrombi, with features of instability, present a higher probability of embolization, justifying surgery as the first therapeutic option in such cases [5].

We report the case of a 48-year-old man with an idiopathic ascending aortic thrombus manifested by acute ischemia of the right upper limb.

## Case presentation

A 48-year-old man, with no relevant personal or family history, presented to the Emergency Department complaining of sudden onset of pain and paresthesias in the right hand and forearm.

On admission, his blood pressure was 160/100 mmHg, with a pulse rate of 70 beats/minute and oxygen saturation of 98% on ambient air. There was no motor deficit of the right upper limb but moderate pain, pallor, poikilothermia, and paresthesias were present in the right hand and forearm. Brachial pulses were palpable and symmetric, while right radial and ulnar pulses were absent. Blood tests were unremarkable. Acute ischemia of the right upper limb was suspected and arterial eco-Doppler confirmed thrombosis of the right brachial artery. The patient was started on anticoagulation with subcutaneous enoxaparin, 1 mg/kg twice daily, with only slight improvement in pain.

**Figure 1:**
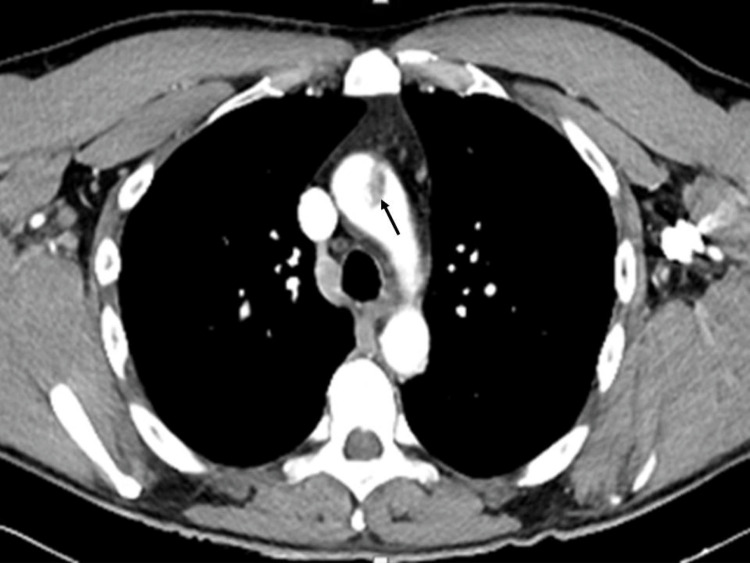
CTA showing an endoluminal repletion defect in the ascending aorta (axial view) CTA: computed tomography angiography

**Figure 2:**
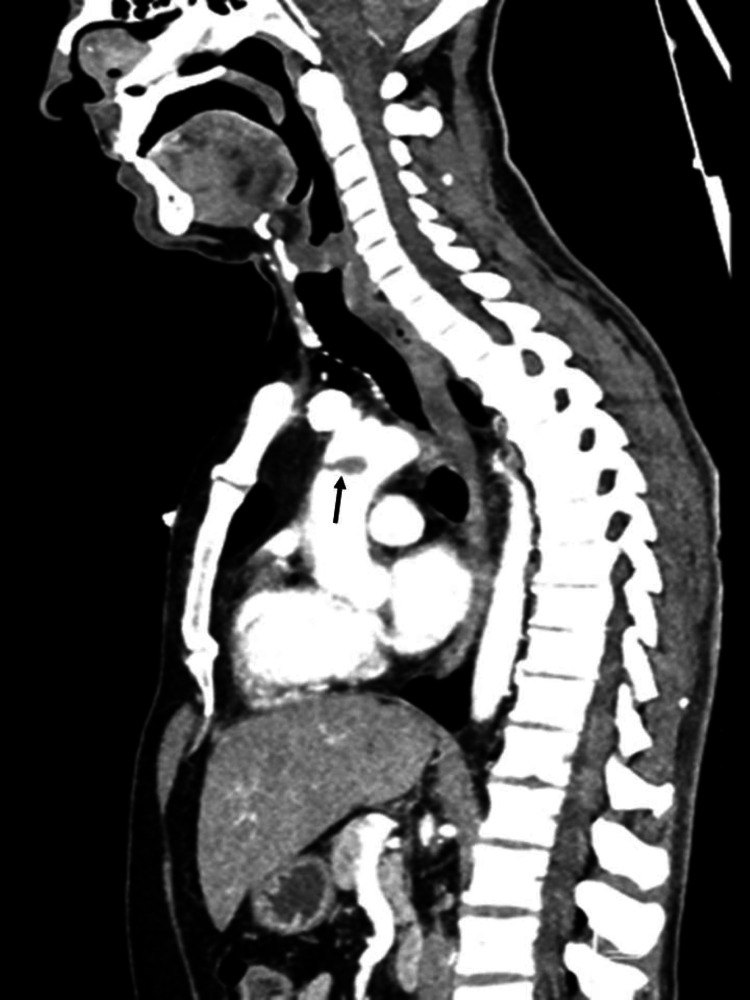
CTA showing an endoluminal repletion defect in the ascending aorta (sagittal view) CTA: computed tomography angiography

The etiological investigation included a computed tomography angiography (CTA) of the thorax, abdomen, and neck that showed an endoluminal repletion defect in the ascending aorta, measuring 2.2x1.2x1.1 cm, adherent to the aortic wall by a millimetric pedicle, compatible with an unstable thrombus, upstream the supra-aortic vessels emergency (Figures 1, 2); no other vascular or extravascular alterations were found. The transthoracic echocardiogram didn´t reveal any left ventricular thrombi or other masses. The laboratory investigation directed to the exclusion of myeloproliferative syndromes and thrombophilias, namely those determining a greater predisposition to arterial thrombosis (hyperhomocysteinemia, antiphospholipid syndrome, and dysfibrinogenemia) were also negative.

**Figure 3:**
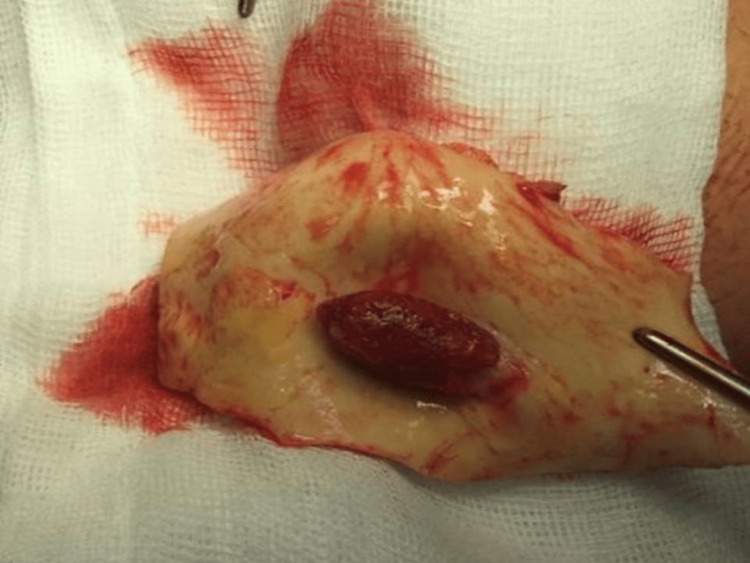
Macroscopic aspect of the excised aortic mass

**Figure 4:**
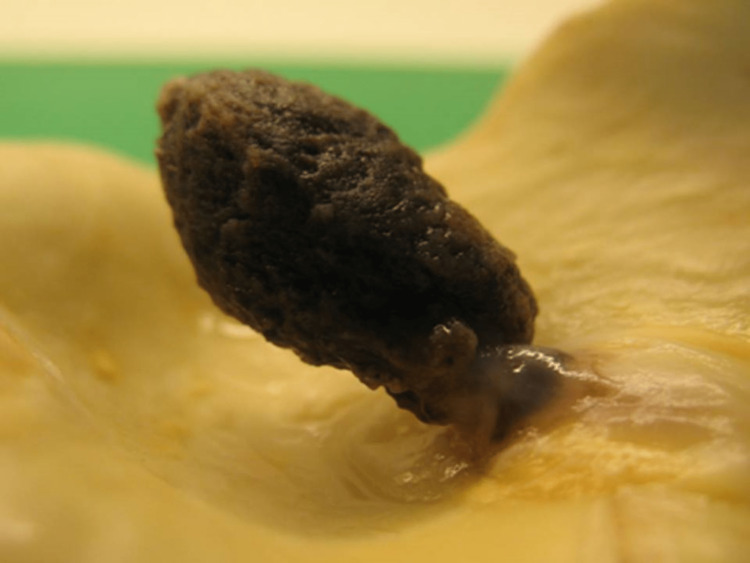
Macroscopic aspect of the excised aortic mass

Facing an imminent risk of new embolic events (including to the central nervous system), dissection, or aortic perforation, the patient underwent urgent surgery to correct the aortic defect and revascularize the right upper limb. The intraoperative transesophageal echocardiogram showed a drop shape mass, pedunculated, in the distal portion of the ascending aorta, adherent to its anterior wall, very mobile (Figures 3, 4); the aorta had a normal caliber in all segments and no signs of dissection or significant atheromatosis (plaques 3 mm thick). The procedure, conducted under profound hypothermia, consisted of excision of the aortic mass and implantation of a tubular prosthesis in the ascending aorta and aortic arch, and thrombo-embolectomy of the right brachial artery. The surgery was uneventful and the patient was discharged after six days on oral anticoagulation.

**Figure 5:**
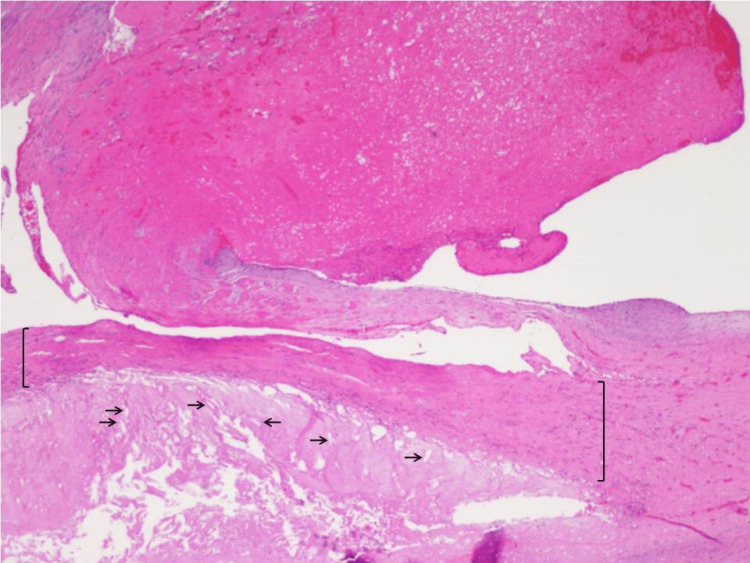
Microscopic aspect of the aortic mass and arterial wall with H&E stain revealing some cholesterol deposits (arrows) and a discrete lymphocytic inflammatory infiltrate (brackets) H&E: hematoxylin and eosin

**Figure 6:**
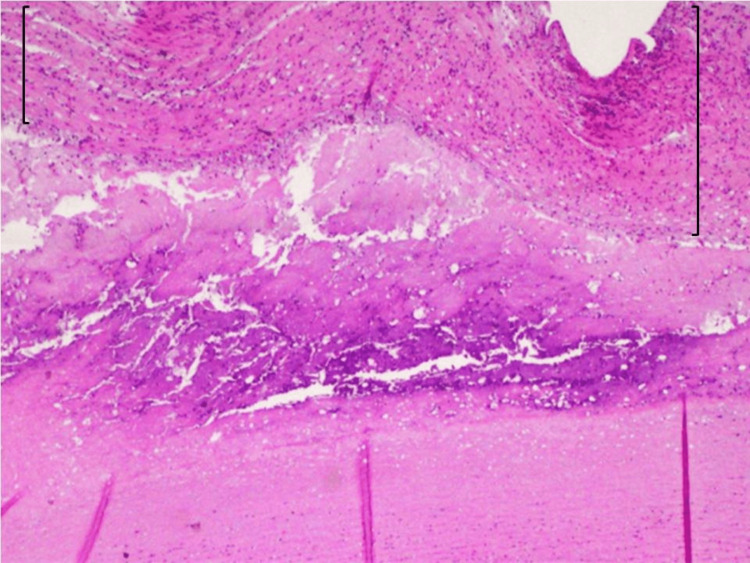
Microscopic aspect of the arterial wall with H&E stain revealing a discrete lymphocytic inflammatory infiltrate (brackets) H&E: hematoxylin and eosin

The histopathological exam of the aorta documented a pedunculated thrombus in organization, with a discrete lymphocytic inflammatory infiltrate in the adjacent vasa vasorum, without vasculitis (Figures 5, 6); the thrombus was adherent to a small/minimal atherosclerotic lesion (expected for the age group); the microbiological exam was negative. The brachial thrombus displayed similar features, confirming embolization. At the nine-month follow-up consultation, the patient was asymptomatic.

## Discussion

Mural thrombi of thoracic aorta are a rare clinical entity, especially in the presence of a non-aneurysmal and non-atherosclerotic vessel [1,4-8]. In some patients, they are idiopathic; however, several etiologies have been described for this pathology. Before classifying an aortic thrombus as primary or idiopathic, it is important to rule out some conditions that predispose to its development: severe atherosclerosis, aneurysmatic or dissecting aortic disease, aortic trauma, neoplasm (either as a paraneoplastic syndrome or direct infiltration of the aortic wall), primary tumors of the aorta, vasculitis (Takayasu's arteritis, giant cell arteritis, and Behçet's disease), and hypercoagulable states, including myeloproliferative syndromes.

Although they are a rare cause of peripheral arterial embolization [1], this is the main manifestation of aorta mural thrombi. A meta-analysis conducted by Fayad et al., which included 200 patients with symptomatic aortic thrombi, found that acute arterial ischemia of lower limbs was its most common embolic manifestation (84%), followed by visceral ischemia (27%) and stroke (14%) [1].

The therapeutic approach to mural aortic thrombi is controversial, mainly due to the low frequency with which they are seen in clinical practice. There are different possible approaches: anticoagulation alone, conventional aortic surgery, thrombolytic therapy, and endovascular treatment. Several factors influence the chosen strategy namely: type and severity of presenting symptoms, age and functional status of the patient, and certain thrombus characteristics such as its location and morphology [6].

Regarding the location, a classification was proposed by Verma et al. [5], which is shown in Table 1.

**Table 1:** Classification of mural thrombi of aorta by location Based on a proposal by Verma et al. [5]

Type	Location
I	Ascending aorta and arch until the left subclavian artery origin
	a) The thrombus is limited to the ascending aorta
	b) The thrombus extends or localizes exclusively in the arch
II	Descending thoracic aorta (distal to left subclavian artery emergency) up to the celiac artery
	a) The thrombus is above T8
	b) The thrombus is between T8-L1
III	Between the celiac artery and the most distal renal artery
IV	Between the lowest renal artery and the aortic bifurcation

Descending thoracic aorta has been reported as the most common segment of aorta in which aortic mural thrombi appear (38%), followed by the arch (36%), abdominal aorta (14%), and, more rarely, ascending aorta (12%) [1]. Type I thrombi are the rarest [5], especially type Ia, which our patient presented. The manifestations of this type of thrombus can be catastrophic since they are located upstream of the emergency of supra-aortic vessels, which means that a greater extent of arterial territories is at risk of embolic events (central nervous system, upper and lower limbs, and visceral).

Regarding the morphology, thrombi can be divided into sessile (eccentric or concentric thrombus with no free floating component); pedunculated (attached to aorta proximally with a distal free-floating segment of variable length), or occlusive [5,6]. Karalis et al. reported a 73% incidence of embolic events in patients with pedunculated and mobile thrombi, in opposition to immobile thrombi with no free-floating component, which had an estimated incidence of 12% [9]. These types of thrombi have been managed in an individualized manner: small sessile thrombi carry a smaller risk of embolization, allowing for anticoagulation alone and close imaging follow-up [9], with surgery reserved for those who fail to respond to the medical strategy or who present recurrent embolization despite the best medical treatment [2,5,8,10,11]. On the other hand, pedunculated and larger thrombi, with instability signs, present a higher risk of embolization and/or its recurrence, and in these cases, surgical excision should be considered, despite the greater morbidity of this approach [5,6,12].

Regarding the therapeutic options, in the systematic review by Fayad et al. that reviewed 98 articles, 112 patients received only anticoagulation and 88 underwent aortic surgery primarily and the conclusions were: 1) the persistence/recurrence of the thrombus was higher in the group subjected only to anticoagulation when compared to the group that underwent aortic surgery (26,4 vs. 5,7%); 2) recurrent embolic episodes were seen in 25,7% of patients in the anticoagulation group and only in 9,1% of those from the surgical group; 3) the mortality rate was similar for both groups (6,2% for the anticoagulation group and 5,7% for the surgical intervention one); 4) the complication rate was higher in the anticoagulation group, namely with amputation rates of 9% vs. 2% in the surgical group as was the need for secondary aortic surgery for persistence/recurrence of the thrombus (24,8% on the anticoagulation group vs. 4,5% on the surgical group) [1]. In the systematic review by Fayad et al., the location of the thrombus in the ascending aorta or arch, the existence of mild atherosclerosis of the arterial wall and the presentation as cerebral ischemia were all important predictors of recurrence of arterial embolic phenomena. These patients are, probably, the ones who benefit the most from aortic surgery as the first approach.

In other small series, 26-27% of the patients, initially treated with anticoagulation alone, presented recurrence of the illness, with the subsequent necessity of secondary aortic surgery [2,13].

Based on the previous data, we can affirm that the treatment of this disease should be individualized and adapted to each patient, with no standard treatment that can be universally applied. Several factors should guide our decision: characteristics, size, and location of the thrombus, as well as clinical presentation, functional status, and surgical conditions of the patient. Only the weighted assessment of all these factors will allow us to obtain the best possible outcome, which means resolution of the thrombus with the lowest morbidity and mortality.

## Conclusions

Although mural thrombi in the ascending portion of the thoracic aorta are rare, their first manifestation as an embolic event is the most frequent scenario and the upper limbs are one of the possible sites of embolization. In face of this finding, it is important to emphasize this entity in the differential diagnosis of acute arterial ischemia of upper limbs. The spectrum of therapeutic interventions can range from anticoagulation alone to conventional surgery or endovascular treatment, based on several factors namely, clinical presentation and individual features of the patient and of the thrombus itself, such as location and morphology.

Even though there are no formal guidelines to standardize approaches, it seems that whenever surgery is possible, the outcomes of these patients are more favorable. Prospective studies to compare the different therapeutic approaches, in specific settings, would be very useful, but the low incidence of this pathology has made it difficult to perform.
